# Uncovering the Ancient Source of Immune System Variety

**DOI:** 10.1371/journal.pbio.0030212

**Published:** 2005-05-24

Animals with adaptive immunity have a secret for deaing with foreign invaders like viruses and bacteria—variety. Their immune systems generate a diverse array of receptors to detect the enormous number of components (antigens) that make up an invader. But with so many potential antigens, it would be difficult for the immune system to anticipate every one and thereby encode a receptor gene for each of them. Instead, the immune system employs a strategy of combinatorial diversity, recombining a few genes to give an unlimited supply of different receptors.

The portions of immune receptor genes that recombine are called V (variable), D (diversity), and J (joining) segments. The immune system randomly recombines these segments in a process called V(D)J recombination. This extraordinary reorganization is undertaken by two enzymes: RAG1 and RAG2. How this process evolved in animals is a mystery, although it has been theorized that RAG1 and RAG2 might have evolved from an ancient enzyme, called transposase, that could move or transpose gene segments. But proof of this theory for the origin of RAG's activity has remained elusive.

In a new study, Vladimir Kapitonov and Jerzy Jurka have found that RAG1 is similar to transposases encoded by transposons (jumping genes that encode transposases necessary for their mobility) found in both terrestrial and marine organisms: the fruit fly and malaria-carrying African mosquito and the sea urchin and hydra. These potentially ancient relatives of RAG1 are all called *Transib* transposons. The discovery of their relation to RAG1 supports the decades-old hypothesis that V(D)J recombination sprung from a transposase.

A number of different types (superfamilies) of transposons exist in nature, but no one has been able to show that RAG1 or RAG2 evolved from them. Kapitonov and Jurka took advantage of the recently discovered *Transib* superfamily of transposons to reexamine this problem. They used seven known *Transib* transposases from the fruit fly and malaria-carrying African mosquito to search the protein database GenBank, finding that part of one *Transib* transposase, *Transib2_AG*, was 35%–38% identical to part of RAG1.

This initial relationship only suggested that RAG1 might be related to *Transib2_AG*, since the similarity between the two was only “marginally” statistically significant, leaving the possibility that it occurred by chance. To find more statistical evidence of a relationship, Kapitonov and Jurka searched for more *Transib* proteins. They found a diverse family of *Transib* transposases in various animals, including silkworm, red flour beetle, dog hookworm, soybean rust, and hydra. The authors also found that plants and vertebrates appear not to contain *Transib* proteins.

With the new proteins in tow, Kapitonov and Jurka found that a 600-amino-acid region of RAG1 was statistically similar to *Transib* transposases. This 600-amino-acid region of RAG1 forms the core region that mediates V(D)J recombination. Three important amino acids, which underlie RAG1's ability to recombine gene segments, are also conserved in *Transib* transposases. Furthermore, RAG1 and RAG2 are known to recombine V, D, and J segments by binding to specific signals in these genes (called recombination signal sequences), which appear to have been derived from the ends of *Transib* transposons. It was previously thought that both RAG1 and RAG2 likely evolved from two proteins encoded by the same transposon. However, Kapitonov and Jurka could not find any RAG2-like proteins encoded by *Transib* transposons. The authors therefore suggest that RAG2 appeared later in jawed vertebrates as a necessary component for the evolution of V(D)J recombination.[Fig pbio-0030212-g001]

**Figure pbio-0030212-g001:**
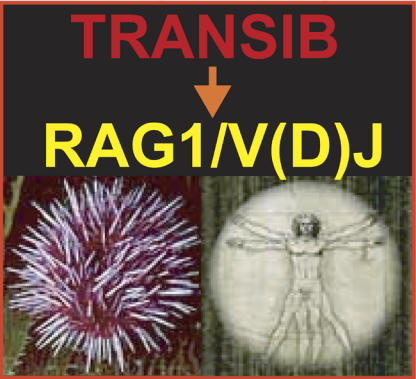
A gene involved in V(D)J recombination—which allows immune cells to recognize an unlimited number of antigens by reshuffling immune receptor gene segments—evolved from an ancient gene-transposing enzyme

With the use of similarity searches (using computer programs to identify comparable parts of proteins and transposons), Kapitonov and Jurka have provided support for the transposon origin of V(D)J recombination. This theory was previously up for debate, as it was possible that RAG1 and RAG2 could have independently evolved to function like transposons. But the authors suggest that “these arguments can now be put to rest,” as it appears RAG1 evolved from a transposon currently found in flies and other organisms. Future experiments on how *Transib* transposons work may allow further understanding into how RAG1 and RAG2 evolved and how they function in vertebrates.

